# Supplementary material for: New Evidence of Tiger Subspecies Differentiation and Environmental Adaptation: Comparison of the Whole Genomes of the Amur Tiger and the South China Tiger
Source: Animals (Basel). 2022 Jul 16;12(14):1817. doi: 10.3390/ani12141817 (PMC9312029; doi:10.3390/ani12141817)
Supplement: Supplementary file 1 [file animals-12-01817-s001.zip › Supplementary Materials.pdf]

1、θπ, θw, Tajima's D ,Fst and Vst statistical method

θπ is defined as:

$$\theta\pi = \sum_{i=1}^{n}\sum_{j=1}^{n} p_i p_j \pi_{ij}$$

$$a_1 = \sum_{i=1}^{n-1} 1/i$$

$$\theta w = S/a_1$$

Where p_i is the frequency of ith sequence, p_j is the frequency of jth sequence, and π_{ij} is the proportion of the difference between sequence i and sequence j. S is Segregating site.

The theoretical value of the population genetic parameter θ was θ=4Neμ, Ne was the effective population size, and μ was the mutation frequency. The Tajima D test was constructed based on the difference between the two estimated values θ w and π.

$$D = \frac{\theta\pi - \theta\omega}{\sqrt{V(\theta\pi - \theta\omega)}}$$

$$Fst = \frac{s^2 - \frac{1}{2\bar{n}-1}\left[\bar{p}(1-\bar{p}) - \frac{r-1}{r}s^2\right]}{\left[1 - \frac{2\bar{n}C^2}{(2\bar{n}-1)r}\right]\bar{p}(1-\bar{p}) + \left[1 + \frac{2\bar{n}(r-1)C^2}{(2\bar{n}-1)r}\right]\frac{s^2}{r}}$$

θπ、θω、Tajima's D and Fst were calculated using NGSTools software package in this study. The genome was slid across a particular size Window (Window size 40KB, step size 20KB), and the population genetic information difference (SNP) in the Sliding Window was analyzed.

$$Vst = \frac{Vt - Vs}{Vt}$$

Where Vt is the variance of all unrelated individual CNV values, Expressed in log2; Vs is the average variance of each population.

2、Quality control procedures of details

Qequence data by Base Calling. The results were stored in fastq file format (file name :*.fq). The raw sequencing data contained "adapter," low-quality bases, and undetected bases (denoted by "N"), which will cause interference to subsequent information analysis. It is removed before analysis and obtained is valuable data, called Clean data or Clean reads. The original data filtering method is as follows:

1, Filter out adapter reads.

2, When N in a Single-read exceeds 10% of the length of the read, then paired reads should be removed

3) When the Single-read contains low quality (<=5), paired reads should be removed if the number of bases exceeds 50% of the read length.

After filtering sequencing data, high-quality Clean data was obtained. The output data of 8 samples were counted, including sequencing data production, sequencing error rate, Q20 content, Q30

content, GC, etc. The total sequencing data volume was 648Gb, and the high-quality clean data volume was 647Gb.
